# Optimization of fermentation parameters for the valorization of tomato pomace into biomass to produce single cell protein using *Saccharomyces cerevisiae*

**DOI:** 10.1186/s40643-026-01115-3

**Published:** 2026-08-17

**Authors:** Ayse Sultan Akgun, Deniz Sevim Cabuk, Dila Cinar, Omer Alp, Dilara Kaya, Nigar Guliyeva, Beste Naz Ince, Defne Gulcek, Mecit Halil Oztop

**Affiliations:** 1 Department of Biotechnology, Graduate School of Natural and Applied Sciences, Middle East Technical University, Ankara, Turkey; 2 Department of Food Engineering, Middle East Technical University, Ankara, Turkey; 3 Center of Excellence in Biomaterials and Tissue Engineering, BIOMATEN, Middle East Technical University, Ankara, Turkey

**Keywords:** Fermentation, Bioprocessing, Tomato pomace, Single cell protein, Optimization, Full factorial design, Second-order polynomial resgression

## Abstract

Tomato pomace, a byproduct of tomato processing industry, can be utilized through fermentation to produce single cell protein (SCP). In this study, fermentation parameters were optimized in a 3-L bioreactor using a full factorial design (FFD), to which a second order polynomial regression model was subsequently fitted to locate the optimum via response surface analysis. While pH, temperature, agitation speed and airflow rate were held constant, the effects of solid load, inoculum volume, and fermentation period were analyzed using a 3 × 3 × 5 experimental design. The resulting biomass was harvested, and its protein content was analyzed. Optimal conditions were identified as a 10.35% solid load and a 2.07% inoculum volume over a 5-day fermentation period. Under these optimized conditions, the model-predicted dry cell weight of 6.92 g/L was validated experimentally at 6.25 g/L, with protein content of 38.40%. These results demonstrate the effective valorization of tomato industry waste into a high value protein resource through optimized bioprocessing.

**Graphical abstract:**

**Figure:**
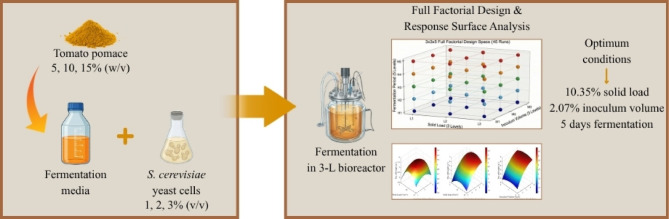

**Supplementary Information:**

The online version contains supplementary material available at 10.1186/s40643-026-01115-3.

## Key points

- Tomato pomace is a valuable source to produce single cell protein.
- Batch fermentation was conducted in 3-L bioreactor.
- Second-order polynomial regression was used to determine optimum parameters.
- Solid load, inoculum volume and fermentation period effects were investigated.

**Supplementary Information:**

The online version contains supplementary material available at 10.1186/s40643-026-01115-3.

## Introduction

Tomato is a widely consumed vegetable and known for containing beneficial components. It is consumed fresh and processed into tomato paste, sauce and juice. The worldwide tomato processing reached 45.8 million tons in 2024 (Morning Star, 2024). The tomato processing industry generates vast amount of tomato pomace mainly consists of peels, seeds and pulp, approximately 3–5% (w/w) (Eslami et al. 2022). Traditional disposal methods, such as landfilling and incineration, are restricted due to carbon emissions and environmental impacts. Therefore, the use of tomato pomace to produce value-added products has become important in terms of need for circular bio-economy strategies.

Tomato pomace is composed of dietary fibers, proteins, lipids, minerals, polyphenols, and carotenoids (Lu et al. 2019; Pirozzi et al. 2022; Ubeyitogullari and Ciftci 2022). Owing to this rich composition, tomato pomace has attracted considerable attention as a valuable raw material for the recovery of various compounds. Previous studies have explored its utilization for the extraction of oil (Arabani et al. 2015; Lisichkov et al. 2011), pectin (Alancay et al. 2017; Grassino et al. 2016), proteins for food and animal feed applications (Aljurf et al. 2026; Baker et al. 2022), bioactive compounds (Concha-Meyer et al. 2020; Kehili et al. 2017), and the development of sustainable biofilms for packaging applications (Simões et al. 2023; Tedeschi et al. 2018).

In addition, the relatively high carbohydrate content of tomato pomace makes it a suitable substrate for fermentation processes, as hydrolysis can generate significant amounts of reducing sugars (Hijosa-Valsero et al. 2019). Its nutrient-rich composition further supports microbial growth and enhances the efficiency of biotechnological processes (Allison et al. 2016; Kehili et al. 2016). Consequently, tomato pomace has been investigated in fermentation-based applications such as the production of alcohols and esters (Kılmanoğlu et al. 2021), the improvement of its nutritional value (Singh et al. 2024), lactic acid production (Paniagua-García et al. 2024), and the generation of bioflavors (Güneşer et al. 2015). Overall, the biochemical composition of tomato pomace makes it a promising substrate for the production of single-cell protein (SCP).

Single-cell protein (SCP) has gained increasing attention as a sustainable protein source due to the growing global population and the associated rise in protein demand. Unlike conventional agricultural protein production, SCP production is largely independent of climatic conditions, soil quality, and land availability (Hülsen et al. 2018). Moreover, SCP exhibits high protein conversion efficiency and provides a wide range of essential nutrients (Matassa et al. 2015). As an alternative protein source, SCP can be produced from agro-industrial wastes through solid-state or submerged fermentation using microorganisms such as bacteria, yeasts, and fungi (Bajić et al. 2022).

For food and feed applications, the use of microorganisms classified as “generally recognized as safe” (GRAS) is essential (Koukoumaki et al. 2024). Among these microorganisms, yeasts are widely used in SCP production due to their rapid growth, high biomass productivity, and ease of harvesting (Koukoumaki et al. 2024). *Saccharomyces cerevisiae* is one of the most commonly used yeast species, traditionally employed in baking and brewing for centuries. Its GRAS status, high nutritional quality, and relatively high protein content (45–60%) make it a suitable candidate for SCP production. In addition, *S. cerevisiae* possesses robust metabolic capabilities that enable the utilization of a wide range of sugars derived from agricultural substrates while remaining low in fat and sodium (Pereira et al. 2021). Compared with other yeast species, it typically contains higher methionine levels but slightly lower lysine levels (Øverland and Skrede 2017).

The composition of yeast biomass can vary depending on the strain, growth medium, and cultivation conditions (Øverland and Skrede 2017). Therefore, maximizing protein yield on complex substrates such as tomato pomace requires careful control and optimization of fermentation conditions.

Fermentation parameters can be precisely controlled using bioreactors operating in batch, fed-batch, or continuous modes. Among these, batch fermentation is the most commonly applied process due to its operational simplicity and ease of handling (Pillaca-Pullo et al. 2025). The yield, quality, physicochemical characteristics, and organoleptic properties of single-cell protein are strongly influenced by the substrate composition and fermentation conditions, including temperature, pH, dissolved oxygen, and agitation speed (Rajput et al. 2024). The use of bioreactors enables stable and reproducible production by maintaining controlled environmental conditions throughout the fermentation process (Simutis and Lübbert 2015). Therefore, optimizing fermentation parameters is essential to maximize SCP yield and product quality.

This study aimed to produce microbial biomass as a sustainable protein alternative by utilizing tomato pomace as a low-cost fermentation substrate. To optimize the bioconversion process, a 3 × 3 × 5 mixed-level full factorial design (FFD) was employed, evaluating three key fermentation parameters: solid load (3 levels), inoculum volume (3 levels), and fermentation period (5 levels), resulting in 45 treatment combinations. Dry cell weight (g/L) was used as the primary response variable, serving as a critical indicator of single-cell protein production efficiency. A second-order polynomial regression model was subsequently fitted to the FFD data to model the individual and interactive effects of the tested parameters on biomass yield and to identify the optimal fermentation conditions. In addition, reducing sugar utilization (%) was monitored throughout fermentation and analyzed in relation to dry cell weight (g/L) to assess substrate conversion efficiency.

## Materials & methods

### Yeast activation and seed culture preparation

The yeast *Saccharomyces cerevisiae* RX60 (Zymaflore RX60, Laffort, France) was cultured in yeast extract–peptone–dextrose (YPD) broth containing 1% yeast extract, 2% peptone, and 2% glucose at 30 °C and 200 rpm for 18 h in an orbital shaking incubator (JSSI-050T, JSR, South Korea) (Olivares-Marin et al. 2018). The grown yeast cells were subsequently streaked onto nutrient agar plates and incubated at 30 °C for 24 h. Colonies were then transferred back into YPD broth and cultivated under the same conditions. The cultures were preserved as stock cultures by mixing with a 50:50 glycerol–water solution and storing at -80 °C for later use.

Prior to fermentation experiments, the growth medium was prepared by adding 50 mL of YPD broth into 250 mL Erlenmeyer flasks, followed by sterilization in an autoclave (FD50A-Uniclave, Zealway, China) at 121 °C for 15 min. The medium was then inoculated with the stock culture and incubated at 30 °C and 200 rpm for 18 h. Cell growth was monitored by measuring the optical density at 600 nm (OD_600_) using a microplate reader (FlexA-200 Microplate Reader, Allsheng, China). The absorbance values were converted to dry cell weight (g/L) using a standard calibration curve prepared for dry cell weights ranging from 0.05 to 0.7 g/L. The standard curve was generated following the procedure described by Mazi (2010).

### Substrate and composition analysis

Dried tomato pomace was obtained from a tomato processing industry after tomato paste production (TAT Gıda Sanayi A.Ş., Bursa, Türkiye). The material was ground using a rotor mill equipped with a 0.5 mm sieve (Pulverisette 16, Fritsch, Germany) and stored in zip-lock bags at 4 °C until further use. Moisture content was determined using an infrared moisture analyzer (MAC 50, Radwag, Poland). Total protein content was analyzed using the Kjeldahl method, applying a nitrogen-to-protein conversion factor of 5.45, which is appropriate for tomato pomace (Aljurf et al. 2026). Fat content was determined by Soxhlet extraction (EFLAB, Türkiye) using hexane as the solvent following the method described by Zhao and Zhang (2013) with slight modifications. Ash content was determined by incineration at 600 °C for 8 h in a muffle furnace (Electro-mag, Türkiye) (Jaafar et al. 2009). Carbohydrate content was calculated by difference to 100%.

### Sample preparation for fermentation

Tomato pomace was used as the carbon source for fermentation. Hydrolysis conditions were selected based on the highest reducing sugar production reported by Akgun et al. (2025). Initially, hydrothermal pretreatment was performed in an autoclave at 121 °C for 15 min. The slurry was then cooled to 50 °C, and enzymatic hydrolysis was carried out by adding 4.24% (v/w) Pectinex Ultra Pulp and 6.20% (v/w) Cellic CTec3 (Novozymes, Denmark). The hydrolysis was performed at 50 °C and 75 rpm for 18 h in a shaking water bath. The resulting hydrolysate was filtered through cheesecloth to remove the bulk insoluble fiber fraction and then centrifuged at 3000 × g for 15 min (NF 1200/1200R, Nuve, Türkiye) to obtain a clarified fermentation medium. Finally, the pH of the medium was adjusted to 5.0 using 1 M NaOH, and the medium was sterilized by autoclaving at 121 °C for 15 min prior to inoculation.

### Fermentation conditions in the bioreactor

Batch fermentation was conducted in a 3 L jacketed bioreactor equipped with a six-bladed Rushton turbine, a sparger, sensors and a control system for temperature, pH, foam detection, alongside a dissolved oxygen (DO) probe for continuous monitoring (Biopex A, Unopex, Türkiye). DO values were observed continuously during each run but were not logged for post-hoc quantitative analysis in the present study. The working volume was set to 1.5 L for each experiment. Temperature, pH, agitation speed, and aeration rate were maintained at 30 °C, pH 5.0, 200 rpm, and 1.2 L/min, respectively. The agitation speed of 200 rpm was selected to minimize shear stress on yeast cells while maintaining adequate mixing. pH was actively maintained at 5.0 throughout the fermentation using an automated controller with 0.2 M NaOH and 0.1 M H_2_SO_4_. An antifoam solution (1:50 dilution) was added to the fermentation medium when necessary. The fermentation process was carried out for 5 days, and samples were collected daily for analysis.

### Experimental design and statistical modeling

A two-step statistical design of experiments (DOE) approach was employed to evaluate the effects of fermentation parameters on dry cell weight (g/L). Initially, a full factorial design (FFD) with a 3 × 3 × 5 mixed-level structure was applied to investigate the effects of three independent variables: solid load (5, 10, and 15% w/v), inoculum volume (1, 2, and 3% v/v), and fermentation period (five levels) as given in Table 1. The experimental design was generated using Minitab software (Version 19, Minitab, UK), resulting in 45 experimental conditions (Table 2). The relatively large number of experimental runs increased the robustness and reliability of the developed statistical model. All experiments were performed in triplicate.

**Table 1:** Experimental factors and levels of the full factorial design for the optimization of biomass production

Factors	Levels
Solid load (%w/v)	5, 10, 15
Inoculum volume (%v/v)	1, 2, 3
Fermentation period (days)	1, 2, 3, 4, 5

**Table 2:** Full factorial experimental design matrix for dry cell weight production

Process factors				Process response
Run	Solid load (%w/v)	Inoculum volume (%v/v)	Fermentation period (day)	Dry cell weight (g/L)
1	5	1	1	1.04 ± 0.30
2	5	1	2	2.23 ± 0.79
3	5	1	3	4.18 ± 1.02
4	5	1	4	5.39 ± 1.10
5	5	1	5	5.21 ± 0.06
6	5	2	1	1.07 ± 0.09
7	5	2	2	3.00 ± 0.06
8	5	2	3	4.09 ± 0.02
9	5	2	4	5.02 ± 0.15
10	5	2	5	4.30 ± 0.17
11	5	3	1	0.62 ± 0.21
12	5	3	2	3.00 ± 0.36
13	5	3	3	4.58 ± 0.20
14	5	3	4	4.40 ± 0.54
15	5	3	5	4.64 ± 0.73
16	10	1	1	1.48 ± 0.80
17	10	1	2	2.67 ± 0.53
18	10	1	3	4.03 ± 0.47
19	10	1	4	5.26 ± 0.51
20	10	1	5	6.06 ± 0.63
21	10	2	1	2.78 ± 0.84
22	10	2	2	5.29 ± 0.97
23	10	2	3	7.02 ± 1.08
24	10	2	4	7.52 ± 0.80
25	10	2	5	6.86 ± 0.87
26	10	3	1	1.08 ± 0.22
27	10	3	2	3.94 ± 0.89
28	10	3	3	5.72 ± 0.84
29	10	3	4	6.49 ± 0.51
30	10	3	5	6.50 ± 0.60
31	15	1	1	1.19 ± 0.21
32	15	1	2	1.71 ± 0.13
33	15	1	3	4.53 ± 0.41
34	15	1	4	5.98 ± 0.50
35	15	1	5	6.38 ± 0.23
36	15	2	1	2.27 ± 0.66
37	15	2	2	2.94 ± 0.54
38	15	2	3	3.77 ± 0.14
39	15	2	4	4.90 ± 0.57
40	15	2	5	5.50 ± 0.69
41	15	3	1	1.38 ± 0.47
42	15	3	2	2.46 ± 0.47
43	15	3	3	3.62 ± 0.78
44	15	3	4	5.29 ± 0.94
45	15	3	5	5.88 ± 0.65

Values are expressed as mean ± SE (*n* = 3)

The factorial design enabled the evaluation of the main effects of the selected variables as well as their potential interactions on dry cell weight. Subsequently, a second-order polynomial regression model was fitted to the experimental data obtained from the factorial design to describe the relationship between the independent variables and the response, and to identify the optimal region for biomass production via response surface analysis. To ensure model parsimony and enhance predictive accuracy, non-significant terms (*p* > 0.05) were removed from the model via a backward elimination process. However, the model hierarchy principle was maintained.

A second-order polynomial model including linear, quadratic, and interaction terms was fitted to the experimental data according to the following equation:

$$\begin{aligned} Y = & \beta _{0} + \sum\limits_{{i = 1}}^{k} {\beta _{i} } x_{i} + \sum\limits_{{i = 1}}^{k} {\beta _{{ii}} } x_{i}^{2} \\ & \quad + \sum\limits_{{i = 1}}^{k} {\sum\limits_{{j = 1}}^{k} {\beta _{{ij}} } } x_{i} x_{j} \\ \end{aligned}$$

where * Y* represents the predicted response (dry cell weight), * k* is the number of independent variables, $\beta _{0}$ is the intercept coefficient, $\beta _{i}$ represents the linear coefficients, $\beta _{{ii}}$ represents the quadratic coefficients, and $\beta _{{ij}}$ represents the interaction coefficients.

The adequacy and significance of the developed model were evaluated using analysis of variance (ANOVA). Table 2.

### Analytical methods

#### Reducing sugar analysis

Samples (1 mL) were collected daily and transferred into microcentrifuge tubes, followed by centrifugation at 9600 × g for 5 min. The supernatant was diluted 30-fold prior to reducing sugar determination using the dinitrosalicylic acid (DNS) method (Miller 1959). Briefly, 3 mL of the diluted sample was mixed with 3 mL of DNS reagent in test tubes and heated at 100 °C for 15 min using a tube heater (Maxtable H10, Daihan, South Korea). The tubes were subsequently cooled in a water bath for 10 min. The absorbance was measured at 575 nm using a microplate reader, and the reducing sugar concentration was calculated as glucose equivalents (g/L) using a calibration curve prepared with glucose standards in the range of 0–1 g/L.

Reducing sugar utilization (%) was calculated using the following equation:

$$\begin{aligned} & {\mathrm{Reducing}}\:{\mathrm{sugar}}\:{\mathrm{utilization}}(\% ) \\ & \quad = {\text{ }}\frac{{C_{{initial}} - C_{{final}} }}{{C_{{initial}} }} \times 100 \\ \end{aligned}$$

where $C_{{initial}}$ is the reducing sugar concentration (g/L) in the fermentation medium before inoculation and $C_{{final}}$ is the reducing sugar concentration (g/L) at the sampling time.

#### Microorganism growth analysis and specific growth rate

To obtain the growth curve of *Saccharomyces cerevisiae*, YPD broth was inoculated with the yeast culture. Subsequently, 0.2 mL of the inoculated culture was transferred into a sterile 96-well microplate and incubated at 30 °C for 22.5 h in a microplate reader. Optical density was measured at 600 nm (OD_600_) at 15 min intervals. Before each measurement, the plate was shaken for 30 s to ensure homogeneous suspension.

To monitor microbial growth during fermentation, optical density measurements were also performed at 600 nm using a microplate reader. For this purpose, 1 mL fermentation samples were centrifuged, and the resulting pellets were washed twice with 1 mL distilled water. The pellets were then resuspended in 1 mL distilled water and diluted 10-fold before measurement. The absorbance values were converted to dry cell weight (g/L) using a calibration curve prepared for dry cell weights ranging from 0.05 to 0.7 g/L.

The specific growth rate (µ) of *S. cerevisiae* during the exponential growth phase was calculated according to the following equation:

$$X\left( t \right) = X_{0} e^{{\mu t}}$$

where $\:X\left(t\right)$ represents the biomass concentration at time * t*, $\:{X}_{0}$ is the initial biomass concentration, and $\:\mu\:\:$ is the specific growth rate. The specific growth rate was determined from the slope of the linear regression of ln(X) versus time during the exponential growth phase.

The biomass doubling time ($\:{t}_{d}$) was calculated using the following equation:

$$\:{t}_{d}=\frac{\mathrm{ln}2}{\mu\:}$$

The biomass yield coefficient ($\:{Y}_{X/S}$) was calculated to evaluate the conversion efficiency of reducing sugars into biomass:

$$X\left( {Y_{{X/S}} = \frac{{\Delta X}}{{\Delta S}}t} \right) = X_{0} e^{{\mu t}}$$

where *Δ X* represents the increase in biomass concentration (g/L) and *Δ S* represents the amount of reducing sugar consumed (g/L).

#### Protein analysis of produced biomass

After 5 days of fermentation, the produced biomass was collected by centrifugation at 3000 × g for 15 min. The harvested biomass was freeze-dried using a lyophilizer (LGJ-10, Songyuan, China) for 36 h, and the final dry biomass weight was recorded. The total protein content of the produced biomass was determined using the Kjeldahl method. A nitrogen-to-protein conversion factor of 5.80 was applied to account for the contribution of non-protein nitrogen sources such as nucleic acids (Anderson et al. 2024). Note that this factor differs from the 5.45 used for raw tomato pomace substrate. The higher factor of 5.80 is standard for yeast biomass to correct for the proportionally greater non-protein nitrogen content in microbial cells.

### Statistical analysis

The experimental data were analyzed using analysis of variance (ANOVA) in Minitab software (Version 19, Minitab, UK). All experiments were performed in triplicate. The results were expressed as mean ± standard error (SE) of at least two independent measurements according to the coefficient of variation. Tukey’s multiple comparison test was used to determine statistically significant differences among groups at a significance level of *p* < 0.05. Significant differences between groups were indicated by different letters.

## Results & discussion

### Tomato pomace composition

The chemical composition of tomato pomace used in this study is presented in Table 3. Although the composition may vary depending on tomato variety and processing conditions, tomato pomace is generally characterized by a high carbohydrate content. This carbohydrate fraction can be hydrolyzed to produce reducing sugars that can subsequently serve as a carbon source during fermentation.

Following hydrolysis under identical conditions, the initial reducing sugar concentrations obtained at different solid loads were determined. The reducing sugar concentrations were found to be 17.60 ± 2.15 g/L, 30.87 ± 1.35 g/L, and 38.23 ± 1.21 g/L for 5, 10, and 15% (w/v) solid loads, respectively. As expected, increasing the solid load resulted in higher reducing sugar concentrations in the hydrolysate. However, the increase is not proportional to solid load due to high solids effect. In lignocellulosic enzymatic hydrolysis, the high-solids effect reduces the hydrolysis rate since free water becomes scarce. Reduced free water increases apparent viscosity and yield stress, which impairs mixing and creates mass and heat transfer limits (Da Silva et al. 2020).

In addition to carbohydrates, the protein present in tomato pomace provides a supplementary nitrogen source, which is beneficial for microbial growth during fermentation. This nutrient composition highlights the potential of tomato pomace as a suitable substrate for microbial biomass production (Paniagua-García et al. 2024).

**Table 3:** Proximate composition analysis of tomato pomace

Composition	% Dry basis
Crude protein	16.21 ± 0.11
Fat	9.88 ± 0.05
Carbohydrate	69.31 ± 0.15
Ash	4.16 ± 0.06

Values are expressed as mean ± SE (*n* = 3)

### Microorganism growth

**Fig. 1:**
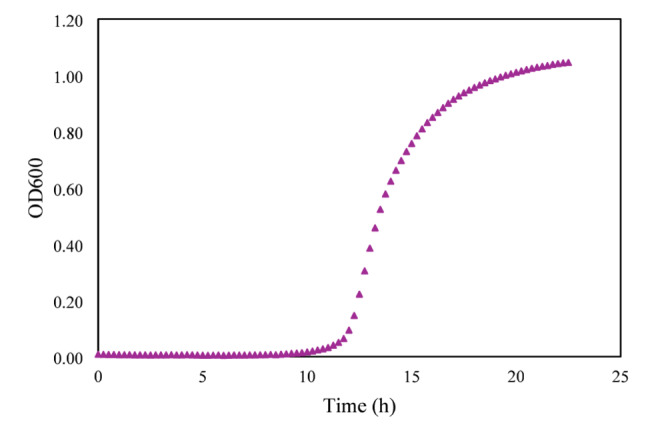
Growth curve of *S. cerevisiae*

The growth profile of *S. cerevisiae* was monitored over a 22.5 h incubation period to determine the optimal physiological state for inoculation (Fig. 1). The growth curve showed that the culture reached the late exponential phase at approximately 18 h. Therefore, this time point was selected as the standardized inoculation time for subsequent fermentation experiments. Harvesting the inoculum during the late exponential phase ensures that the yeast cells exhibit high metabolic activity and viability, which are critical for efficient fermentation initiation. The maximum specific growth rate (µ_max_) of *S. cerevisiae* during the exponential phase was calculated as 1.084 h^−1^. This relatively high growth rate indicates strong metabolic activity and rapid adaptation of the strain under the cultivation conditions used in this study (Bruggeman et al. 2023).

### Effects of the input variables on dry cell weight and growth kinetics

**Fig. 2:**
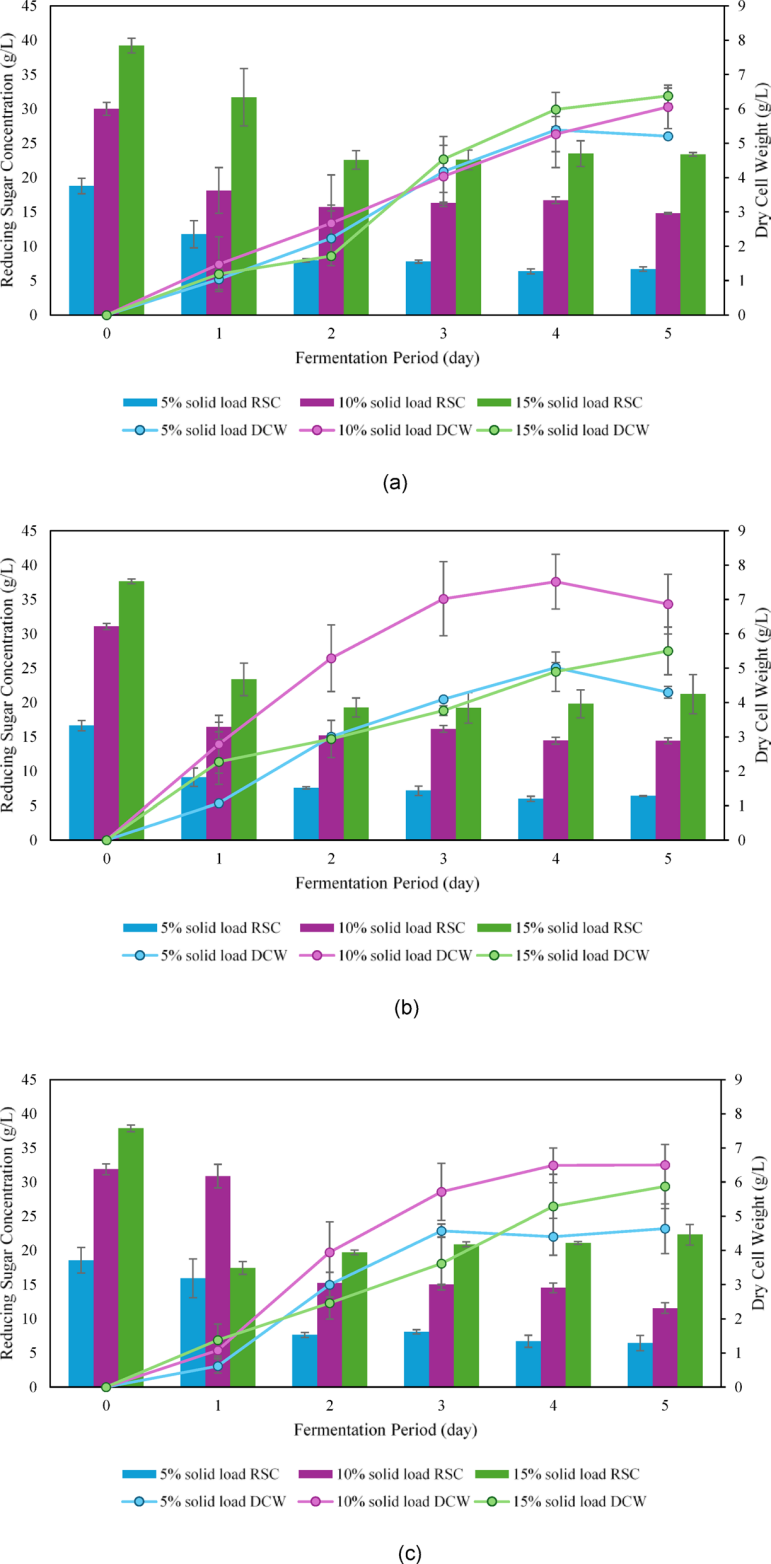
Changes in reducing sugar concentration and dry cell weight during fermentation under different solid loads, at **(a)** 1%, **(b)** 2%, and **(c)** 3% (v/v) inoculum volume

The dry cell weight obtained under different fermentation conditions is presented in Table 2. The dynamic relationship between reducing sugar concentration and biomass production during fermentation is illustrated in Fig. 2. Figure 2a, b, and c correspond to inoculum volumes of 1, 2, and 3% (v/v), respectively.

As fermentation progressed, reducing sugar concentrations gradually decreased while dry cell weight increased, indicating active substrate consumption and biomass formation. After approximately day 2–3 of fermentation, reducing sugar concentrations began to stabilize particularly at the 5 and 10% solid loads as shown in Fig. 2, whereas biomass production continued to increase until reaching its maximum between day 4 and day 5. This observation suggests that yeast growth was not solely dependent on the initially available reducing sugars.

The continued increase in biomass despite the stabilization of reducing sugar concentration may be partly explained by a diauxic shift (Kottelat et al. 2021). During the first 48 h of fermentation, *S. cerevisiae* rapidly consumes the readily available reducing sugars in the fermentation medium, primarily glucose and fructose, leading to rapid biomass formation. Once these preferred carbon sources become limited, the yeast undergoes metabolic reprogramming and begins to utilize alternative substrates. In this secondary growth phase, ethanol produced during the initial fermentative metabolism, as well as other metabolites such as organic acids and glycerol, may serve as alternative carbon sources supporting further cell growth and protein synthesis (Mazzoleni et al. 2015).

In addition, yeast cells may metabolize non-reducing or initially undetected carbohydrates present in the hydrolysate. For instance, non-reducing disaccharides such as sucrose can be hydrolyzed intracellularly and subsequently utilized for respiration (Verhagen et al. 2025). Furthermore, hydrolysates derived from lignocellulosic substrates often contain mixtures of pentose and hexose sugars. While *S. cerevisiae* efficiently metabolizes hexoses, pentose utilization is generally limited due to carbon catabolite repression mechanisms (Kim et al. 2010; Saxena et al. 2023). Therefore, the residual reducing sugars detected in the fermentation medium may largely consist of pentose sugars that are not readily metabolized by the yeast.

A similar relationship between sugar utilization and biomass formation has been reported in previous studies. For example, Yang et al. (2025) observed an increase in single-cell protein content from 32.5 to 36.0% as fermentable sugar utilization increased in a co-culture fermentation system.

Among the tested conditions, the highest dry cell weight (7.52 g/L) was obtained after 4 days of fermentation using 10% solid load and 2% inoculum volume. It should be noted that this single point observation exceeds the later model predicted optimum of 6.92 g/L. This is expected, as the polynomial model represents a smoothed surface fitted across all 135 data points rather than a direct interpolation of individual observations. The model optimum therefore reflects the most consistently achievable maximum across replicates, not the highest single measurement. Although increasing the solid load increased the initial reducing sugar concentration, it did not proportionally accelerate biomass formation, as shown in Fig. 2b and c.

The calculated specific growth rate, biomass doubling time, and biomass yield are presented in Table 4. A higher specific growth rate generally indicates faster biomass formation and SCP accumulation. However, substrate utilization does not always result in efficient biomass or protein production. Under certain conditions, metabolic pathways that support higher specific growth rates may lead to lower biomass yields, whereas pathways associated with higher yields often result in slower growth rates. This trade-off between growth rate and yield may occur under oxygen-limited conditions, where metabolic flux is redirected toward fermentative pathways rather than biomass synthesis (Wortel et al. 2018). Real-time observation of dissolved oxygen during fermentation indicated a decline from initial saturation (approximately 100%) to a minimum of 3–5% during the period of peak biomass accumulation, with this decline consistently more pronounced at higher solid loads and inoculum volumes than at lower ones, before partial recovery (to 25–30%) as growth slowed toward the end of fermentation.

In addition, growth rate can influence the biochemical composition of microbial biomass. Lower specific growth rates are often associated with higher intracellular protein fractions and altered amino acid profiles, while higher growth rates favor rapid biomass accumulation but may result in increased RNA content and lower protein concentration per cell (Sakarika et al. 2023). These observations highlight that fermentation performance depends not only on biomass production but also on the physiological state and metabolic regulation of the microorganism. Notably, the biomass yield coefficient (Y_X/S_) did not differ significantly across any of the tested solid load and inoculum volume combinations (*p* > 0.05), suggesting that the overall substrate-to-biomass conversion efficiency remained relatively stable regardless of the initial conditions applied. The invariance of Y_X/S_ across the design space indicates that metabolic efficiency remained constant regardless of solid load or inoculum volume. Consequently, variations in dry cell weight were driven by the total fermentable substrate available rather than changes in microbial efficiency. This suggests that biomass productivity in this system is scaled primarily by substrate input, while manipulations of inoculum volume or solid-to-inoculum ratios do not alter the underlying conversion yields.

**Table 4:** Specific growth rate, doubling time and yield under interactions of solid load and inoculum volume

Solid load (%w/v)	Inoculum volume (%v/v)	Specific growth rate (µ, day^− 1^)	Doubling time (t_d_, day)	Yield (Y_X/S_, g/g)
5	1	0.47 ± 0.06^ab^	1.49 ± 0.18^abc^	0.55 ± 0.03^a^
5	2	0.71 ± 0.00^ab^	0.97 ± 0.00^bc^	0.42 ± 0.01^a^
5	3	0.99 ± 0.30^a^	0.77 ± 0.23^c^	0.53 ± 0.04^a^
10	1	0.42 ± 0.06^ab^	1.68 ± 0.25^abc^	0.39 ± 0.10^a^
10	2	0.31 ± 0.04^b^	2.28 ± 0.29^a^	0.35 ± 0.01^a^
10	3	0.81 ± 0.01^ab^	0.86 ± 0.01^c^	0.32 ± 0.02^a^
15	1	0.70 ± 0.04^ab^	0.99 ± 0.05^bc^	0.50 ± 0.12^a^
15	2	0.55 ± 0.13^ab^	1.33 ± 0.31^abc^	0.28 ± 0.05^a^
15	3	0.33 ± 0.05^b^	2.18 ± 0.45^ab^	0.33 ± 0.07^a^

Values are expressed as mean ± SE (*n* = 2). Lowercase superscript letters (a–c) denote a significant difference at 5% (*p* < 0.05) in each column

### Statistical analysis of the effects of fermentation parameters on dry cell weight

The experimental results for dry cell weight (g/L) obtained from the 3 × 3 × 5 factorial design were subjected to second-order polynomial regression to evaluate the influence of solid load (X_1_), inoculum volume (X_2_), and fermentation period (X_3_). Initially, Full Factorial Design (FFD) regression analysis was performed to identify the significant factors affecting biomass production. The analysis revealed that solid load and fermentation period were highly significant (*p* < 0.001), and inoculum volume was significant (*p* = 0.031). Among the interaction terms, only the interaction between solid load and inoculum volume (*p* = 0.001) was found to be significant, whereas the other interaction effects were not significant. It should be noted, however, that these significant values reflect a first-order polynomial model. When a second-order polynomial is subsequently fitted, the linear terms for solid load and inoculum volume may lose significance as their quadratic terms absorb the curvature-driven variance.

Following FFD regression analysis, a second-order polynomial regression model was fitted to the experimental data to further characterize and optimize the fermentation process. The resulting second-order polynomial regression equation describing the relationship between the independent variables and dry cell weight is given below:

$$\begin{aligned} {\mathrm{Dry}}\:{\mathrm{Cell}}\:{\mathrm{Weight}}\:({\mathrm{g/L}}){\text{ = }} & - {\mathrm{7}}.{\text{14 + 0}}.{\mathrm{964X}}_{{\mathrm{1}}} \\ & {\text{ + 2}}.{\mathrm{168X}}_{{\mathrm{2}}} {\text{ + 2}}.{\mathrm{637X}}_{{\mathrm{3}}} \\ & - {\mathrm{0}}.{\mathrm{04651X}}_{{\mathrm{1}}}^{{\mathrm{2}}} \\ & - {\mathrm{0}}.{\mathrm{523X}}_{{\mathrm{2}}}^{{\mathrm{2}}} \\ & - {\mathrm{0}}.{\mathrm{2546X}}_{{\mathrm{3}}}^{{\mathrm{2}}} \\ \end{aligned}$$

where $\:{X}_{1}$, $\:{X}_{2}$, and $\:{X}_{3}$ represent solid load, inoculum volume, and fermentation period, respectively.

The analysis of variance (ANOVA) results indicate that the developed second-order polynomial model is highly significant (*p* < 0.001) with a non-significant lack-of-fit (*p* = 0.146), as presented in Table 5. Both the linear (*p* < 0.001) and quadratic (*p* < 0.001) components of the model significantly contributed to the response, indicating that the relationship between the fermentation variables and dry cell weight exhibits a significant curvature.

The model explained 72.45% of the variability in dry cell weight (R^2^ = 0.7245). Furthermore, the predicted R^2^ (69.44%) was in good agreement with the adjusted R^2^ (71.16%), indicating satisfactory predictive capability and model reliability. Model adequacy in the present study is supported by four independent criteria. First, the regression model itself was highly significant (*p* < 0.001). Second, the lack of fit test was non-significant (*p* = 0.146), indicating that the second-order polynomial captures the systematic structure of the data and that the residual variance does not exceed pure error from replicate runs. Third, the three coefficients of determination are mutually consistent, with a gap of less than 2% between R^2^ and adj-R^2^ and a gap of approximately 2% between adj-R^2^ and pred-R^2^ which explains that the model is neither over-parameterized nor over-fitted. Fourth, the validation experiment under the model predicted optimum returned a dry cell weight of 6.25 g/L against a predicted 6.92 g/L, corresponding to a 9.68% deviation that lies well within the dispersion typically observed across independent fermentation runs in this system. The remaining unexplained variance likely reflects inherent biological variability across the runs including inoculum-to-inoculum differences and minor fluctuations in the complex tomato pomace substrate composition.

The non-significant linear terms for solid load and inoculum volume indicate that neither factor had a simple directional effect on biomass production within the studied range. Rather, intermediate levels of both variables were most favorable, confirming the existence of an optimum within the experimental domain and justifying the use of a second-order model to locate it.

**Table 5:** Analysis of variance of experimental results of second order polynomial regression analysis

Source	DF	Sum of squares	Mean square	F-value	*p*-value
Model	6	408.673	68.112	56.10	0.000
X_1_	1	2.555	2.555	2.10	0.149
X_2_	1	0.502	0.502	0.41	0.521
X_3_	1	332.343	332.343	273.75	0.000
X_1_^2^	1	40.559	40.559	33.41	0.000
X_2_^2^	1	8.220	8.220	6.77	0.010
X_3_^2^	1	24.495	24.495	20.18	0.000
Lack-of-fit	38	55.517	1.461	1.32	0.146
Pure error	90	99.878	1.110		

The statistical analysis revealed that solid load exhibited a clear second-order effect on dry cell weight. While the initial linear screening suggested a direct influence, the polynomial regression model demonstrated that the relationship between solid load and biomass production is quadratic (*p* < 0.001). The noticeable decline in biomass production when the solid load increased from 10 to 15% (w/v) indicates that the fermentation process is sensitive to substrate overloading. High substrate concentrations can cause inhibitory effects during fermentation. Similar observations have been indicated by Yang et al. (2022), who reported significantly lower biomass production at elevated organic concentrations.

The protein content and biomass formation in microbial fermentation are closely related to nutrient availability. Increasing the tomato pomace concentration may provide additional carbohydrates, nitrogen, vitamins, and minerals that support microbial growth. However, excessive substrate concentrations may lead to substrate inhibition. In complex substrates such as tomato pomace, *S. cerevisiae* may redirect a substantial portion of the consumed sugars toward cellular maintenance rather than biomass synthesis under high solid-load conditions (Moreno et al. 2019).

Regarding inoculum volume, although the linear regression term was not statistically significant (*p* = 0.521), differences among the tested levels were observed. An inoculum volume of 2% (v/v) resulted in the highest dry cell weight (7.52 g/L). Increasing the inoculum volume from 1 to 2% (v/v) significantly improved biomass production (*p* = 0.023), whereas a further increase to 3% (v/v) did not provide additional gains. High inoculum concentrations can shorten the lag phase but may also introduce inhibitory effects due to excessive cell density. Conversely, low inoculum levels often result in prolonged lag phases before exponential growth begins, which can delay protein synthesis and reduce overall productivity (Jalasutram et al. 2013).

Another possible explanation for the reduced biomass production at higher inoculum levels is oxygen limitation. High cell densities increase oxygen demand during fermentation, which can lead to oxygen transfer limitations (Verbelen et al. 2009). Since the aeration rate was kept constant in the present study, oxygen availability may have become limiting at higher inoculum levels, resulting in lower biomass formation.

The fermentation period was identified as the most influential linear factor affecting biomass production (*p* < 0.001). According to the second-order polynomial regression model, dry cell weight increased progressively with fermentation time and reached its maximum between days 4 and 5. Earlier fermentation stages resulted in significantly lower biomass concentrations. Similar observations have been reported in previous studies. For example, Tropea et al. (2022) reported the highest crude protein content (40.19%) after 120 h of fermentation when mixed food waste was used as substrate for *S. cerevisiae*. However, no significant increase was observed after 72 h, and prolonged fermentation eventually resulted in a decrease in protein content due to yeast cell death.

In another study, Saravanan et al. (2023) reported a maximum biomass concentration of 4.56 g/L with a protein content of 27.9% after 48 h fermentation using a 10% (v/v) inoculum. Similarly, Razzaq et al. (2020) reported that optimal SCP production was achieved on the fifth day of fermentation, while extending the fermentation period beyond this point did not significantly increase SCP yield.

The significant quadratic terms in the regression model provide further insight into the metabolic constraints of the system. The negative coefficients for the squared terms of solid load (*p* < 0.001), inoculum volume (*p* = 0.010), and fermentation period (*p* < 0.001) indicate that the response surfaces are concave, suggesting the presence of distinct optimal conditions within the experimental range. The strong quadratic effect of solid load, despite its non-significant linear effect (*p* = 0.149), suggests that biomass production increases up to a threshold level before being inhibited by excessive substrate concentrations. Similarly, the curvature observed for fermentation time reflects the transition from exponential growth to the stationary phase, where biomass accumulation gradually stabilizes.

These results highlight the value of polynomial regression applied to FFD data, as the optimal conditions for maximum dry cell weight occur within the experimental range rather than at the extreme levels of the factors.

**Fig. 3:**
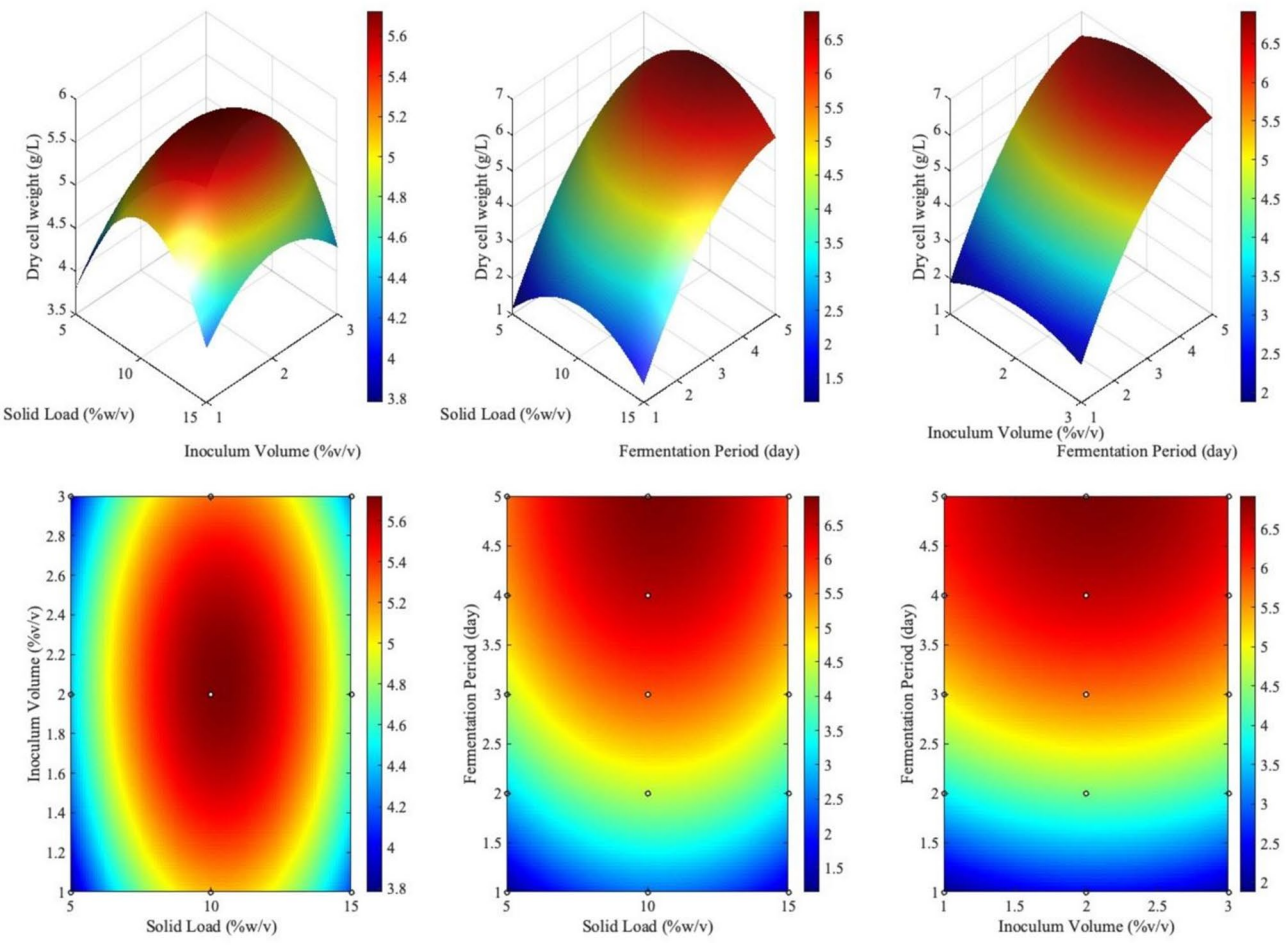
Response surface and contour plots for dry cell weight representing effects of factors

The three-dimensional response surface plots and contour plots illustrating the interactions between the independent variables are presented in Fig. 3. When the solid load was kept constant, dry cell weight increased with increasing inoculum volume up to an optimal point and subsequently decreased. Similarly, increasing the solid load increased dry cell weight up to approximately 10% (w/v), after which further increases resulted in reduced biomass production.

When the fermentation period was increased, dry cell weight increased rapidly, confirming that fermentation time has a strong influence on biomass accumulation. In contrast, the effect of inoculum volume was comparatively less pronounced. These surface plots confirm that fermentation period is the most influential factor affecting dry cell weight, while inoculum volume plays a secondary role in the system.

### Response optimization and model validation

Response surface optimizer in Minitab (Version 19) was employed to determine the optimal fermentation parameters for maximizing dry cell weight production. The model predicted the optimal conditions as 10.35% (w/v) solid load, 2.07% (v/v) inoculum volume, and a fermentation period of 5 days, corresponding to a predicted dry cell weight of 6.92 g/L.

It should be noted that the model-predicted optimum for fermentation period sits on the upper boundary of the experimental range (5 days). Preliminary fermentation studies were conducted at intermediate conditions (10% solid load and 2% inoculum volume) to determine fermentation period boundaries. The dry cell weight was tracked until day 7; biomass was observed to decrease after day 5, indicating that the active growth phase had ended and that further extension of the fermentation did not yield additional cell mass. On the basis of this preliminary observation, the upper level of the fermentation-period factor in the 3 × 3 × 5 design was set at day 5, which therefore represents the practical onset of stationary phase rather than an arbitrary truncation of the design space. The model-predicted optimum landing on this boundary is therefore a confirmation, not an artifact, of the design choice. The decline observed beyond day 5 in the preliminary experiment is consistent with cell autolysis, depletion of fermentable carbon sources, and progressive accumulation of inhibitory fermentation end-products such as ethanol once the active growth phase has ended.

To validate the reliability of the developed model, fermentation experiments were conducted under the predicted optimal conditions. The experimentally obtained dry cell weight was 6.25 g/L, corresponding to a percent error of 9.68% relative to the model predicted value of 6.92 g/L. This deviation is likely attributable to scale dependent effects and batch-to-batch variability inherent in complex biological systems. The 48.80% reducing sugar utilization observed at the optimal conditions indicates that approximately half of the reducing sugars present in the hydrolysate remained unconsumed at the end of fermentation due to limited pentose uptake and oxygen transfer rate redirecting metabolic flux toward fermentative ethanol formation and thereby reducing the rate of net hexose consumption during the late phase of the fermentation. In lignocellulosic or fruit cell wall substrates, xylose remains unused or accumulates (Salafia et al. 2022; Tang et al. 2025). The study conducted by Zhang et al. (2026) showed strain engineering is necessary to consume xylose in the fermentation by *S. cerevisiae*. The crude protein content of the produced biomass was found as 38.40%. The relatively close agreement between the predicted and experimental values confirms the adequacy of the developed response surface model for describing biomass production in the studied system.

### Protein content of the produced biomass

The biomass production and crude protein content of the produced biomass under different fermentation conditions are presented in Table 6. Solid load had a significant effect on biomass production. Fermentations with 5% solid load yielded significantly lower biomass (5.77–6.62 g) compared to those at 10 and 15% solid loads (10.11–13.98 g), as indicated by distinct letter groupings (*p* < 0.05). Within the 10 and 15% solid load groups, increasing inoculum volume from 1 to 3% did not result in statistically significant differences in biomass production. The highest biomass yield (13.98 ± 0.97 g) was recorded at 15% solid load with 3% inoculum volume.

Regarding crude protein content, the fermentation at 5% solid load with 3% inoculum volume yielded the highest protein content (41.75 ± 0.51%), which was significantly higher than treatments at 10% solid load with 1% inoculum volume (37.28 ± 0.61%) and 15% solid load with 1% inoculum volume (36.71 ± 0.18%) (*p* < 0.05). All remaining fermentations were statistically intermediate, sharing letter groups with both extremes.

The protein content of microbial biomass is strongly influenced by nutrient availability in the fermentation medium (Mujdalipah and Putri 2020). Increasing tomato pomace concentration provides additional carbohydrates, nitrogen sources, vitamins, and minerals that support microbial growth and protein synthesis. However, the observed decline in protein content at higher solid loads despite increased total biomass suggests that substrate inhibition or metabolic stress may limit efficient protein synthesis at elevated substrate concentrations. Furthermore, although total biomass may continue to increase with prolonged fermentation, the protein fraction of the biomass may not increase proportionally due to nitrogen depletion and cellular metabolic adjustment during later fermentation stages (Dunuweera et al. 2021; Zhang et al. 2025).

**Table 6:** Interaction effects of solid load and inoculum volume on *S. cerevisiae* fermentation performance

Solid load (%w/v)	Inoculum volume (%v/v)	Reducing sugar utilization (%)	Biomass produced (g)	Crude protein (%)
5	1	58.13 ± 0.09^ab^	5.82 ± 0.06^c^	39.37 ± 1.53^ab^
5	2	61.06 ± 2.47^ab^	5.77 ± 0.27^c^	39.79 ± 1.11^ab^
5	3	56.37 ± 2.21^abc^	6.62 ± 0.32^bc^	41.75 ± 0.51^a^
10	1	55.42 ± 0.11^abc^	10.11 ± 0.13^ab^	37.28 ± 0.61^b^
10	2	54.16 ± 2.34^abc^	12.42 ± 1.61^a^	39.13 ± 0.70^ab^
10	3	66.11 ± 4.22^a^	11.80 ± 0.68^a^	40.61 ± 2.05^ab^
15	1	41.72 ± 1.42^c^	11.50 ± 0.35^a^	36.71 ± 0.18^b^
15	2	49.02 ± 4.40^bc^	12.04 ± 0.12^a^	37.97 ± 0.33^ab^
15	3	48.34 ± 3.23^bc^	13.98 ± 0.97^a^	39.56 ± 0.38^ab^

Values are expressed as mean ± SE (*n* = 2). Lowercase superscript letters (a-c) denote a significant difference at 5% (*p* < 0.05) in each column

## Conclusion

This study investigated the effects of key fermentation parameters on biomass production and protein content using tomato pomace hydrolysate as a substrate for single-cell protein production. The reducing sugars obtained from tomato pomace were successfully utilized as a carbon source, demonstrating the potential of converting tomato processing waste into a valuable alternative protein resource that may contribute to reducing the environmental burden of the food industry.

The application of a full factorial experimental design followed by second-order polynomial regression enabled systematic optimization of the fermentation process. The results indicated that moderate levels of solid load and inoculum volume promote biomass production during fermentation. The optimal fermentation conditions were determined as 10.35% (w/v) solid load, 2.07% (v/v) inoculum volume, and a fermentation period of 5 days, corresponding to a predicted dry cell weight of 6.92 g/L. Experimental validation under these conditions yielded a dry cell weight of 6.25 g/L with 38.40% crude protein content, confirming the adequacy of the developed model.

Overall, the findings demonstrate that tomato pomace can serve as a promising substrate for sustainable single-cell protein production. Future studies should explore specific strategies to further enhance biomass yield and protein content, including dissolved oxygen control to prevent oxygen limitation at high cell densities, supplementary nitrogen feeding with ammonium sulfate to sustain protein synthesis during the stationary phase, and cascade or fed-batch fermentation architectures to extend the productive growth window beyond the constraints of batch operation.

## Supplementary Information

Below is the link to the electronic supplementary material.

Supplementary Material 1

## Data Availability

Data will be made available on request.

## References

[CR1] Akgun AS, Mentes D, Oztop MH (2025) Valorization of tomato pomace to use it as a substrate in the fermentation. Acta Hort 1445:295–302. 10.17660/ActaHortic.2025.1445.41

[CR2] Alancay MM, Lobo MO, Quinzio CM, Iturriaga LB (2017) Extraction and physicochemical characterization of pectin from tomato processing waste. J Food Meas Charact 11(4):2119–2130. 10.1007/s11694-017-9596-0

[CR3] Aljurf BN, Cutroneo S, Bugatti K, Barlas B, Bashir AKH, Sahin S, Oztop HM, Tedeschi T (2026) Tomato pomace as a sustainable protein source: comprehensive characterization of proteins extracted by conventional alkaline, microwave, and ultrasound-assisted methods. Food Hydrocolloids 176:112565. 10.1016/j.foodhyd.2026.112565

[CR4] Allison BJ, Cádiz JC, Karuna N, Jeoh T, Simmons CW (2016) The effect of ionic liquid pretreatment on the bioconversion of tomato processing waste to fermentable sugars and biogas. Appl Biochem Biotechnol 179(7):1227–1247. 10.1007/s12010-016-2061-4 27039400 10.1007/s12010-016-2061-4

[CR5] Anderson A, Van Der Mijnsbrugge A, Cameleyre X, Gorret N (2024) From yeast screening for suitability as single cell protein to fed-batch cultures. Biotechnol Lett. 10.1007/s10529-024-03504-0 39002086 10.1007/s10529-024-03504-0

[CR6] Arabani AA, Hosseini F, Abbaspour F, Anarjan N (2015) The effects of ultrasound pretreatment processes on oil extraction from tomato wastes. Int J Biosci 4(4):8–15. 10.12692/ijb/4.4.8-15

[CR7] Bajić B, Vučurović D, Vasić Đ, Jevtić-Mučibabić R, Dodić S (2022) Biotechnological production of sustainable microbial proteins from agro-industrial residues and by-products. Foods 12(1):107. 10.3390/foods12010107 36613323 10.3390/foods12010107 PMC9818480

[CR8] Baker PW, Preskett D, Krienke D, Runager KS, Hastrup ACS, Charlton A (2022) Pre-processing waste tomatoes into separated streams with the intention of recovering protein: towards an integrated fruit and vegetable biorefinery approach to waste minimization. Waste Biomass Valoriz 13(8):3463–3473. 10.1007/s12649-022-01748-3

[CR9] Bruggeman FJ, Teusink B, Steuer R (2023) Trade-offs between the instantaneous growth rate and long‐term fitness: consequences for microbial physiology and predictive computational models. BioEssays 45(10):2300015. 10.1002/bies.202300015 10.1002/bies.202300015 37559168

[CR10] Concha-Meyer A, Palomo I, Plaza A, Gadioli Tarone A, Junior MRM, Sáyago-Ayerdi SG, Fuentes E (2020) Platelet Anti-Aggregant activity and bioactive compounds of ultrasound-assisted extracts from whole and seedless tomato pomace. Foods 9(11):1564. 10.3390/foods9111564 33126732 10.3390/foods9111564 PMC7694063

[CR11] Da Silva AS, Espinheira RP, Teixeira RSS, De Souza MF, Ferreira-Leitão V, Bon EPS (2020) Constraints and advances in high-solids enzymatic hydrolysis of lignocellulosic biomass: a critical review. Biotechnol Biofuels 13(1):58. 10.1186/s13068-020-01697-w 32211072 10.1186/s13068-020-01697-w PMC7092515

[CR12] Dunuweera AN, Nikagolla DN, Ranganathan K (2021) Fruit waste substrates to produce single-cell proteins as alternative human food supplements and animal feeds using baker’s yeast (*Saccharomyces cerevisiae*). J Food Qual 2021:1–6. 10.1155/2021/9932762

[CR13] Eslami E, Carpentieri S, Pataro G, Ferrari G (2022) A comprehensive overview of tomato processing by-product valorization by conventional methods versus emerging technologies. Foods 12(1):166. 10.3390/foods12010166 36613382 10.3390/foods12010166 PMC9818577

[CR14] Grassino AN, Brnčić M, Vikić-Topić D, Roca S, Dent M, Brnčić SR (2016) Ultrasound assisted extraction and characterization of pectin from tomato waste. Food Chem 198:93–100. 10.1016/j.foodchem.2015.11.095 26769509 10.1016/j.foodchem.2015.11.095

[CR15] Güneşer O, Demirkol A, Karagül Yüceer Y, Özmen Toğay S, İşleten Hoşoğlu M, Elibol M (2015) Bioflavour production from tomato and pepper pomaces by Kluyveromyces marxianus and Debaryomyces hansenii. Bioprocess Biosyst Eng 38(6):1143–1155. 10.1007/s00449-015-1356-0 25614449 10.1007/s00449-015-1356-0

[CR16] Hijosa-Valsero M, Garita-Cambronero J, Paniagua-García AI, Díez-Antolínez R (2019) Tomato waste from processing industries as a feedstock for biofuel production. Bioenergy Res 12(4):1000–1011. 10.1007/s12155-019-10016-7

[CR17] Hülsen T, Hsieh K, Lu Y, Tait S, Batstone DJ (2018) Simultaneous treatment and single cell protein production from agri-industrial wastewaters using purple phototrophic bacteria or microalgae—a comparison. Bioresour Technol 254:214–223. 10.1016/j.biortech.2018.01.032 29413925 10.1016/j.biortech.2018.01.032

[CR18] Jaafar RA, Rahman ARBA, Mahmod NZC, Vasudevan R (2009) Proximate analysis of dragon fruit (Hylecereus polyhizus). Am J Appl Sci 6(7):1341–1346. 10.3844/ajassp.2009.1341.1346

[CR19] Jalasutram V, Kataram S, Gandu B, Anupoju GR (2013) Single cell protein production from digested and undigested poultry litter by Candida utilis: optimization of process parameters using response surface methodology. Clean Technol Environ Policy 15(2):265–273. 10.1007/s10098-012-0504-3

[CR20] Kehili M, Schmidt LM, Reynolds W, Zammel A, Zetzl C, Smirnova I, Allouche N, Sayadi S (2016) Biorefinery cascade processing for creating added value on tomato industrial by-products from Tunisia. Biotechnol Biofuels 9(1):261. 10.1186/s13068-016-0676-x 27980671 10.1186/s13068-016-0676-x PMC5133755

[CR21] Kehili M, Kammlott M, Choura S, Zammel A, Zetzl C, Smirnova I, Allouche N, Sayadi S (2017) Supercritical CO_2_ extraction and antioxidant activity of lycopene and β-carotene-enriched oleoresin from tomato (*Lycopersicum esculentum L*.) peels by-product of a Tunisian industry. Food Bioprod Process 102:340–349. 10.1016/j.fbp.2017.02.002

[CR22] Kim J-H, Block DE, Mills DA (2010) Simultaneous consumption of pentose and hexose sugars: an optimal microbial phenotype for efficient fermentation of lignocellulosic biomass. Appl Microbiol Biotechnol 88(5):1077–1085. 10.1007/s00253-010-2839-1 20838789 10.1007/s00253-010-2839-1 PMC2956055

[CR23] Kılmanoğlu H, Hoşoğlu Mİ, Güneşer O, Yüceer YK (2021) Optimization of pretreatment and enzymatic hydrolysis conditions of tomato pomace for production of alcohols and esters by Kluyveromyces marxianus. LWT 138:110728. 10.1016/j.lwt.2020.110728

[CR24] Kottelat J, Freeland B, Dabros M (2021) Novel strategy for the calorimetry-based control of fed-batch cultivations of* Saccharomyces cerevisiae*. Processes 9(4):723. 10.3390/pr9040723

[CR25] Koukoumaki DI, Tsouko E, Papanikolaou S, Ioannou Z, Diamantopoulou P, Sarris D (2024) Recent advances in the production of single cell protein from renewable resources and applications. Carbon Resour Convers 7(2):100195. 10.1016/j.crcon.2023.07.004

[CR26] Lisichkov K, Kuvendziev S, Lisichkov B (2011) Isolation of tomato seed oil from tomato waste by application of supercritical fluid CO_2_ extraction. Qual Life (Banja Luka) - APEIRON. 10.7251/QOL1101005L

[CR27] Lu Z, Wang J, Gao R, Ye F, Zhao G (2019) Sustainable valorisation of tomato pomace: a comprehensive review. Trends Food Sci Technol 86:172–187. 10.1016/j.tifs.2019.02.020

[CR28] Matassa S, Batstone DJ, Hülsen T, Schnoor J, Verstraete W (2015) Can direct conversion of used nitrogen to new feed and protein help feed the world?. Environ Sci Technol 49(9):5247–5254. 10.1021/es505432w 25816205 10.1021/es505432w

[CR29] Mazi BG (2010) Evaluating Microemulsions for Purification of Beta-Galactosidase from Kluyveromyces lactis [Middle East Technical University]. http://etd.lib.metu.edu.tr/upload/12612692/index.pdf

[CR30] Mazzoleni S, Landi C, Cartenì F, De Alteriis E, Giannino F, Paciello L, Parascandola P (2015) A novel process-based model of microbial growth: self-inhibition in* Saccharomyces cerevisiae* aerobic fed-batch cultures. Microb Cell Fact 14(1):109. 10.1186/s12934-015-0295-4 26223307 10.1186/s12934-015-0295-4 PMC4518646

[CR31] Miller GL (1959) Use of dinitrosalicylic acid reagent for determination of reducing sugar. Anal Chem 31(3):426–428. 10.1021/ac60147a030

[CR32] Moreno AD, González-Fernández C, Ballesteros M, Tomás-Pejó E (2019) Insoluble solids at high concentrations repress yeast’s response against stress and increase intracellular ROS levels. Sci Rep 9(1):12236. 10.1038/s41598-019-48733-w 31439886 10.1038/s41598-019-48733-w PMC6706384

[CR33] Morning Star (2024) Post Season Global Tomato Crop Update. https://www.morningstarco.com/2024-post-season-global-tomato-crop-update/ (accessed 20 January 2026).

[CR34] Mujdalipah S, Putri ML (2020) Utilization of pineapple peel and rice washing water to produce single cell proteins using* Saccharomyces cerevisiae*. IOP Conf Series: Earth Environ Sci 472(1):012029. 10.1088/1755-1315/472/1/012029

[CR35] Olivares-Marin IK, González-Hernández JC, Regalado-Gonzalez C, Madrigal-Perez LA (2018)* Saccharomyces cerevisiae* exponential growth kinetics in batch culture to analyze respiratory and fermentative metabolism. J Visualized Experiments. 10.3791/58192 10.3791/58192 PMC6235381 30320748

[CR36] Øverland M, Skrede A (2017) Yeast derived from lignocellulosic biomass as a sustainable feed resource for use in aquaculture. J Sci Food Agric 97(3):733–742. 10.1002/jsfa.8007 27558451 10.1002/jsfa.8007

[CR37] Paniagua-García AI, Garita-Cambronero J, González-Rojo S, Díez-Antolínez R (2024) Optimization of lactic acid production from apple and tomato pomaces by thermotolerant bacteria. J Environ Manage 366:121806. 10.1016/j.jenvman.2024.121806 39003899 10.1016/j.jenvman.2024.121806

[CR38] Pereira PR, Freitas CS, Paschoalin VMF (2021) *Saccharomyces cerevisiae* biomass as a source of next-generation food preservatives: evaluating potential proteins as a source of antimicrobial peptides. Compr Rev Food Sci Food Saf 20(5):4450–4479. 10.1111/1541-4337.12798 34378312 10.1111/1541-4337.12798

[CR39] Pillaca-Pullo OS, Lopes M, Bautista‐Cruz A, N., Estela‐Escalante W (2025) From coffee waste to nutritional gold: bioreactor cultivation of single‐cell protein from *Candida sorboxylosa*. J Chem Technol Biotechnol 100(2):360–368. 10.1002/jctb.7778

[CR40] Pirozzi A, Ferrari G, Donsì F (2022) Cellulose isolation from tomato pomace pretreated by high-pressure homogenization. Foods 11(3):266. 10.3390/foods11030266 35159418 10.3390/foods11030266 PMC8833915

[CR41] Rajput SD, Pandey N, Sahu K (2024) A comprehensive report on valorization of waste to single cell protein: strategies, challenges, and future prospects. Environ Sci Pollut Res 31(18):26378–26414. 10.1007/s11356-024-33004-7 10.1007/s11356-024-33004-7 38536571

[CR42] Razzaq ZU, Khan MKI, Maan AA, Rahman SU (2020) Characterization of single cell protein from* Saccharomyces cerevisiae* for nutritional, functional and antioxidant properties. J Food Meas Charact 14(5):2520–2528. 10.1007/s11694-020-00498-x

[CR43] Sakarika M, Kerckhof F-M, Van Peteghem L, Pereira A, Van Den Bossche T, Bouwmeester R, Gabriels R, Van Haver D, Ulčar B, Martens L, Impens F, Boon N, Ganigué R, Rabaey K (2023) The nutritional composition and cell size of microbial biomass for food applications are defined by the growth conditions. Microb Cell Fact 22(1):254. 10.1186/s12934-023-02265-1 38072930 10.1186/s12934-023-02265-1 PMC10712164

[CR44] Salafia F, Ferracane A, Tropea A (2022) Pineapple waste cell wall sugar fermentation by* Saccharomyces cerevisiae* for second generation bioethanol production. Fermentation 8(3):100. 10.3390/fermentation8030100

[CR45] Saravanan K, Rajendran M, Kathirvel P (2023) Utilization of water hyacinth (Eichhornia crassipes) as a feasible substrate for the production of single cell protein using* Saccharomyces cerevisiae* (Baker’s Yeast). Waste Biomass Valoriz 14(12):4231–4242. 10.1007/s12649-023-02138-z

[CR46] Saxena A, Hussain A, Parveen F, Ashfaque M (2023) Current status of metabolic engineering of microorganisms for bioethanol production by effective utilization of pentose sugars of lignocellulosic biomass. Microbiol Res 276:127478. 10.1016/j.micres.2023.127478 37625339 10.1016/j.micres.2023.127478

[CR47] Simões A, Coelhoso IM, Alves VD, Brazinha C (2023) Recovery and purification of cutin from tomato by-products for application in hydrophobic films. Membranes 13(3):261. 10.3390/membranes13030261 36984648 10.3390/membranes13030261 PMC10059779

[CR48] Simutis R, Lübbert A (2015) Bioreactor control improves bioprocess performance. Biotechnol J 10(8):1115–1130. 10.1002/biot.201500016 26228573 10.1002/biot.201500016

[CR49] Singh J, Dwivedi B, Kumar V (2024) Optimization of solid-state fermentation conditions for enhancing the nutrition contents in tomato pomace using RSM. J Institution Eng (India): Ser A 105(2):409–418. 10.1007/s40030-024-00783-8

[CR50] Tang C, Martín C, Jönsson LJ (2025) Effects of aeration of softwood pretreatment liquid on inhibitors and fermentability using* Saccharomyces cerevisiae* yeast. Biotechnol Biofuels Bioprod 18(1):103. 10.1186/s13068-025-02708-4 41063163 10.1186/s13068-025-02708-4 PMC12505720

[CR51] Tedeschi G, Benitez JJ, Ceseracciu L, Dastmalchi K, Itin B, Stark RE, Heredia A, Athanassiou A, Heredia-Guerrero JA (2018) Sustainable fabrication of plant cuticle-like packaging films from tomato pomace agro-waste, beeswax, and alginate. ACS Sustain Chem Eng 6(11):14955–14966. 10.1021/acssuschemeng.8b03450

[CR52] Tropea A, Ferracane A, Albergamo A, Potortì AG, Turco L, V., Di Bella G (2022) Single cell protein production through multi food-waste substrate fermentation. Fermentation 8(3):91. 10.3390/fermentation8030091

[CR53] Ubeyitogullari A, Ciftci ON (2022) Enhancing the bioaccessibility of lycopene from tomato processing byproducts via supercritical carbon dioxide extraction. Curr Res Food Sci 5:553–563. 10.1016/j.crfs.2022.01.020 35309261 10.1016/j.crfs.2022.01.020 PMC8928129

[CR54] Verbelen PJ, Saerens SMG, Van Mulders SE, Delvaux F, Delvaux FR (2009) The role of oxygen in yeast metabolism during high cell density brewery fermentations. Appl Microbiol Biotechnol 82(6):1143–1156. 10.1007/s00253-009-1909-8 19263049 10.1007/s00253-009-1909-8

[CR55] Verhagen KJA, Pardijs IH, Van Klaveren HM, Wahl SA (2025) A dive into yeast’s sugar diet—comparing the metabolic response of glucose, fructose, sucrose, and maltose under dynamic feast/famine conditions. Biotechnol Bioeng 122(4):1035–1050. 10.1002/bit.28935 39865609 10.1002/bit.28935 PMC11895419

[CR56] Wortel MT, Noor E, Ferris M, Bruggeman FJ, Liebermeister W (2018) Metabolic enzyme cost explains variable trade-offs between microbial growth rate and yield. PLoS Comput Biol 14(2):e1006010. 10.1371/journal.pcbi.1006010 29451895 10.1371/journal.pcbi.1006010 PMC5847312

[CR57] Yang R, Chen Z, Hu P, Zhang S, Luo G (2022) Two-stage fermentation enhanced single-cell protein production by Yarrowia lipolytica from food waste. Bioresour Technol 361:127677. 10.1016/j.biortech.2022.127677 35878768 10.1016/j.biortech.2022.127677

[CR58] Yang YQ, Li X, Wang ZZ, Huang XY, Zeng DW, Zhao XQ, Liu ZQ, Zhang FL (2025) Single cell protein production of co-culture Kodamaea ohmeri and* Lactococcus lactis* in corn straw hydrolysate. Bioresour Technol 433:132649. 10.1016/j.biortech.2025.132649 40409427 10.1016/j.biortech.2025.132649

[CR59] Zhang X, Zhang H, Wang R, Zhang Z, Yan X, Zhang J (2025) Bioconversion of Baijiu Huangshui for single cell protein using Saccharomyces cerevisiae: a study on influence of carbon metabolic pathways. J Environ Manag 393:127077. 10.1016/j.jenvman.2025.127077 40845647 10.1016/j.jenvman.2025.127077

[CR60] Zhang P, He Z, Li H, Jiang Z (2026) Resurrection and characterization of ancestral xylose transporters enhance the capability of xylose uptake in the mixed sugar co-fermentation of Recombinant* Saccharomyces cerevisiae*. Bioresources Bioprocess 13(1):1. 10.1186/s40643-025-00995-1 10.1186/s40643-025-00995-1 PMC12770209 41489796

[CR61] Zhao S, Zhang D (2013) A parametric study of supercritical carbon dioxide extraction of oil from Moringa oleifera seeds using a response surface methodology. Sep Purif Technol 113:9–17. 10.1016/j.seppur.2013.03.041

